# Preparation and Comparative Bioavailability Studies of Indomethacin-Loaded Cetyl Alcohol Microspheres

**DOI:** 10.1155/2013/109837

**Published:** 2012-09-19

**Authors:** N. Vishal Gupta, D. V. Gowda, V. Balamuralidhara, M. S. Khan

**Affiliations:** Department of Pharmaceutics, JSS College of Pharmacy, JSS University, Sri Shivarathreeshwara Nagar, Karnataka, Mysore 570015, India

## Abstract

The purpose of the present study was to compare the *in vitro* release and to find out whether the bioavailability of a 75 mg indomethacin capsule (Microcid SR) was equivalent to optimized formulation (indomethacin-loaded cetyl alcohol microspheres). Indomethacin-loaded cetyl alcohol microspheres were prepared by meltable emulsified cooling-induced technique. Surface morphology of microspheres has been evaluated using scanning electron microscopy. A single dose, randomized, complete cross over study of IM microspheres was carried out on 10 healthy male and female Albino sheep's under fasting conditions. The plasma was separated and the concentrations of the drug were determined by HPLC-UV method. Plasma indomethacin concentrations and other pharmacokinetic parameters obtained were statistically analyzed. The SEM images revealed the spherical shape of fat microspheres, and more than 98.0% of the isolated microspheres were in the size range 12–32 *μ*m. DSC, FTIR spectroscopy and stability studies indicated that the drug after encapsulation with fat microspheres was stable and compatible. Both formulations were found to be bioequivalent as evidenced by *in vivo* studies. Based on this study, it can be concluded that cetyl alcohol microspheres and Microcid SR capsule are bioequivalent in terms of the rate and extent of absorption.

## 1. Introduction

 In recent years, various uses of wax and fat microspheres in the pharmaceutical field have come into forefront, involving the microspheres technology [1]. The goal of any drug delivery system is to provide a therapeutic amount of drug(s) to the proper site in the body in order to promptly achieve and thereby to maintain the desired drug concentrations during treatment. This idealized objective can be achieved by targeting the drugs to a specific organ or tissue with the help of controlling the release rate of the drug during the transit time in gastrointestinal tract. Poorly water-soluble drugs, which are lipophilic in nature mix, easily with fat and show good absorption rate. Among the reported conventional methods different strategies have been developed in recent years to design different types of wax microspheres loaded with hydrophilic and lipophilic drugs using toxic solvents. The use of such solvents during formulation is of environmental concern and also faces challenge to human safety. To overcome these problems, in the present investigation, water is used to prepare wax microspheres by meltable dispersed emulsified cooling-induced solidification method. Furthermore, the process was optimized to produce microspheres to give better yield with spherical geometry and predictable dissolution pattern.

 Cetyl alcohol (CA), used in the current study, has good pharmaceutical and biological properties [2]. CA is hard, but oily to the touch, and is devoid of taste or smell, making it very useful as an ingredient in cosmetics, as a pharmaceutical excipient. It is an innocuous material generally regarded as essentially nontoxic and nonirritant, biodegradable, biocompatible, nonimmunogenic, gastroresistant, of high carrier capacity, and having controlled release of drug, low production costs, reproducible properties, and good shelf life. But the hypersensitivity reported in some cases may be due to impurities in commercial grades of CA. In pharmaceutical formulations, it is used in the preparation of suppositories, delayed release solid dosage forms, emulsions, lotions, creams, and ointment. Regulatory status: it is included in the FDA as inactive ingredient and in nonparenteral medicines licensed in the UK [3].

IM is a nonsteroidal, anti-inflammatory agent with antipyretic, analgesic properties and is an indole derivative designated chemically as 1-(p-chlorobenzoyl)-5-methoxy-2-methyl-1H-indole-3-acetic acid. IM is an odorless, pale yellow to yellow tan crystalline substance. It is lipid-soluble, practically insoluble in water and sparingly soluble in alcohol. IM has a pKa of 4.5 and is stable in neutral or slightly acidic media and decomposes in strong alkali. The suspension has a pH of 4.0–5.0 and it has a melting point between 155°C and 161°C and has molecular weight of 357.8. IM has a molecular formula of C_18_H_16_ClN0_4_ [4]. Nowadays IM is widely used in the treatment of active stages of moderate-to-severe stages of rheumatoid arthritis. IM should be dosed at least 2–3 times per day. Due to its narrow therapeutic index, the frequency of adverse effects is dosing related [5]. Considering the long therapeutic regimen of osteoarthritis therapy, the administration of IM may induce adverse side effects on gastrointestinal tract (GIT) as well as central nervous system (CNS), renal and cardiac systems [6]. The occurrence of these adverse effects can be reduced by the use of controlled release formulations [7]. Oral conventional dosage forms are administered 2-3 times a day to maintain adequate and effective therapeutic concentration in blood; however, it fails to protect the patients against morning stiffness [8].

Development of controlled release formulation of IM has several advantages over the other conventional dosage forms, such as reduction in occurrence of high initial peak plasma concentrations, protection against morning stiffness, prolonged duration of action, improved bioavailability, patient compliance and reduction in adverse effects [9]. The side effects could be lowered by controlling the drug release and by adjusting the absorption rate. This can be achieved by employing suitable modification in the manufacturing process [10]. Previous experimental results have demonstrated that the waxes are biocompatible, nonimmunogenic material used for the entrapment of drug and its controlled drug release in the intestinal tract [11]. Delivering the drug in the intestinal environment from fatty CA microspheres could be manipulated by suitable coating techniques [12]. The chief characteristics of enteric coating are their impermeability to gastric juice, but susceptibility to intestinal juice [13, 14]. Desired plasma levels can be achieved without the risk of side effects using once-a-day dose of controlled release preparation [15]. These findings suggested that the kinetic control is an effective route for preventing the toxicity of IM.

 The aim of the present study was to formulate, characterize, and study the *in vitro* release of IM from microspheres and compare with commercially available oral formulation Microcid SR (75 mg capsule) furthermore, to investigate the pharmacokinetics and bioavailability of two different oral IM formulation (optimized microsphere formulation and Microcid SR 75 mg capsule) following a single dosing in healthy Albino sheeps in order to prove the bioequivalence between the preparations.

## 2. Materials and Methods 

### 2.1. Materials

 Indomethacin (IM) and mefanamic acid (MA), the internal standard, were kindly donated by Micro Labs (Bangalore, India). Cetyl alcohol (CA-Melting point 49–51°C), Tween 80, and all other chemicals and solvents used were of analytical grade and purchased from Ranbaxy Fine chemicals (New Delhi, India). Commercially available oral capsule formulation (Microcid SR 75 mg, Micro Labs Ltd., India) was used for the present study.

### 2.2. Methods

#### 2.2.1. Preparation of Microspheres

9 gm of CA was melted in a china dish kept on water bath. To the melted wax mixture, IM (3 gm) previously passed through sieve no. 100 was dispersed in melted wax mass and stirred to obtain a homogeneous mixture. The resultant mixture was then poured into 150 mL of phthalate buffer solution (pH 4.5), previously heated to a temperature higher than melting point of CA (>50°C). The surfactant, Tween 80 (0.3% w/w), was added to the above mixture and stirred mechanically at 900 rpm using a stirrer (RQ-127A, Remi, India). Spherical particles were produced due to dispersion of molten CA in the aqueous medium. The mixture was stirred continuously above the melting point of CA at 900 rpm for 5 min. The temperature of the reaction mixture was cooled rapidly and brought down to 10°C by the addition of cold water. The resultant solid spheres were collected by filtration and washed with water to remove surfactant residue. Air-drying was carried out at room temperature for 48 h to give discrete, solid, and free flowing microspheres. A total of five formulations were prepared by varying the CA to drug ratios (Table 1).

#### 2.2.2. Microsphere Characterization

Tap density of the prepared CA microspheres was determined using tap density tester, and percentage Carr's index (% I) was calculated using the formula:

(1) Carr's  index(%  I)  =[(tapped  density−bulk  density)tapped  density]×100.

Angle of repose (*h*) was assessed to know the flow ability of CA microspheres, by a fixed funnel method:

(2) Tan(θ)=heightradius.

#### 2.2.3. Scanning Electron Microscopic Studies and Sphericity Determination

Scanning electron microscope (SEM) photomicrographs were recorded using Joel-LV-5600 SEM, USA. Sphericity of microspheres was determined using an image analysis system. Photomicrographs were taken with a digital camera (Sony, DSC T-4010. Cyber shot, Japan). The obtained images were processed by image analysis software to characterize each individual microsphere by mean Feret diameter (FD) (average of 180 caliper measurements with an angle of rotation of 1°), Aspect ratio (AR) (ratio of longest Feret diameter and its longest perpendicular diameter), and two-dimensional shape factor (*eR*) by the following equation:

(3) eR=2πrPm−  (bl)2,

where *r* is the radius, Pm is the perimeter, *l* is length (longest Feret diameter), and *b* is width (longest perpendicular diameter to the longest Feret diameter) of the microspheres.

#### 2.2.4. Differential Scanning Calorimetry (DSC)

All dynamic DSC studies were carried out on DuPont thermal analyzer with 2010 DSC module. Calorimetric measurements were made with the help of an empty cell (high purity alpha alumina discs of DuPont Company) as the reference. The instrument was calibrated using high purity indium metal as standard. The dynamic scans were taken in nitrogen atmosphere at the heating rate of 10°/min. The runs were made in triplicate.

#### 2.2.5. Fourier Transforms Infrared Spectroscopy (FTIR)

FTIR spectra of pure drug, empty microspheres, and drug-loaded microspheres were obtained using KBr pellet method (applying 6000 kg/cm^2^). Spectral measurements were obtained by powder diffuse reflectance on an FTIR spectrophotometer (Shimadzu, FTIR 8400S, Japan) in the wave number region of 400–4000 cm^−1^ to study drug excipient interactions.

#### 2.2.6. Estimation of Drug Loading

Drug incorporated CA microspheres of each batch were selected and powdered in a mortar. Drug was extracted from CA microspheres using methanol, filtered and analyzed for drug content after suitable dilution. Estimation of IM was accomplished by UV/Visible spectroscopy (Shimadzu–1601, Japan) at 319 nm after sufficient dilution with pH 7.2 phosphate buffer.

#### 2.2.7. *In Vitro* Release Studies

 USP XXII dissolution apparatus type II was employed to study percentage of drug release from various formulations prepared. Accurately weighed quantities of drug-loaded microspheres (IM-equivalent to 75 mg) of each batch were taken in 900 mL dissolution medium (2 h in pH 1.2 hydrochloric acid buffer and 6 h in pH 7.2 phosphate buffer) and stirred at 100 rpm by maintaining at a temperature of 37  ±  0.5°C. The drug concentrations were determined by withdrawing the 10 mL of aliquots using guarded sample collectors periodically at an interval of 30 min for first 4 h and at 60 min interval for the next 4 h. Release studies were carried out in triplicate.

#### 2.2.8. Stability Studies

The optimized formulation was subjected for stability studies and stored in glass bottles at 25°C/60% RH (relative humidity), 30°C/65% RH, and 40°C/75% RH for a period of 90 days. 100 mg of microspheres from each batch of formulations was taken at the end of 30th, 60th, and 90th days and was subjected for drug content studies.

#### 2.2.9. *In Vivo* Studies

*In vivo* release studies have been conducted on five male and five female healthy adult albino sheeps. The sheep ages were in the range 5–7 years and their body weight ranged between 25 and 28 kg. A written approval was obtained from the Institutional Ethical Committee of JSS College of Pharmacy, Mysore, India. Detailed verbal and written information on the study was provided to the Veterinary Surgeon, Central Animal Facility, JSS Medical College Hospital, and permission was obtained. The study was conducted as an open, randomized complete crossover design in which a single 75 mg dose of IM (Microcid SR 75 mg capsule and formulation F_3_) was administered to fasted, healthy adult males and females on two different occasions, separated by a wash-out period of 2 weeks between dosing interval. The content uniformity of marketed product and optimized formulation has been estimated as per USP specification [16].

The contents of 5 units of Microcid SR 75 mg capsule and formulation F_3_ were individually combined and weighed to the average weight of each unit. Drug was extracted from the respective dosage forms using methanol (80%). Methanolic extract was suitably diluted and drug content was determined.

All the animals were shifted to the clinical trial laboratory from animal house at 6.00 AM after overnight fast of 10 h. After shaving near the neck, an 18 gauge (1.3  ×  45 mm, 96 mL/min) cannula was inserted into a jugular vein and kept with heparinised saline lock for ensuing 24 h blood sampling. Test medications (marketed product and optimized formulation) were administered to the sheeps, fed with banana and 200 mL water. Light food was provided at 3rd h followed by two standard meals at 7th and 11th h following drug administration. Blood samples (5 mL) were collected at 0 h (pre dose interval) and at 0.5, 1, 1.5, 2, 2.5, 3, 3.5, 4, 5, 6, 7, 8, 12, 16, 20, and 24 h postdose intervals. Blood samples were centrifuged (eltek TC 4100 D centrifuge, Elektroshaft, India) at 1500 rpm for 10 min. The separated plasma was stored at −20°C prior to analysis. Any other type of food was not permitted after 12 h administration of test medication. All subjects remained ambulatory and strenuous physical activity was prohibited during the first 12 h of blood sampling. Plasma concentration of drug from the collected samples was quantified by modified HPLC method [17].

#### 2.2.10. Extraction Procedure

Internal standard Mefanamic acid (MA) (100 *μ*L) and citrate buffer (pH 3.0, 500 *μ*L) were added to 10 mL screw capped glass tubes containing 500 *μ*L of spiked plasma. The tubes were extracted gently with 7 mL of petroleum ether:dichloromethane (50 : 50) for 5 min on a rotary shaker and centrifuged at 900 rpm for 5 min. The organic phase was transferred to a watch glass and evaporated to dryness at 40°C. The residue was resuspended in 100 *μ*L of mobile phase and 25 *μ*L was injected to the column. Quantification was achieved by the measurement of the peak area ratio of the IM to the internal standard (mefanamic acid). The limit of detection of IM in plasma was 100 ng/mL (500 *μ*L of plasma injected) [14]. 

#### 2.2.11. Chromatographic Studies

The IM concentrations in plasma were assayed using a fully validated high performance liquid chromatography with ultraviolet detection (HPLC-UV) method [16], with respect to adequate sensitivity, specificity, linearity, recovery, accuracy, and precision (both within and between days). The HPLC system consisted of HPLC-Shimadzu (Tokyo, Japan) LC-6A model, fitted with a *μ*-Bondapack C18 (4.6 × 250 mm) column of particle size 5 *μ*m (Supelco, Bellefonte, PA). The flow rate was maintained at 1 mL/min, and the drug concentration was detected using a UV/visible detector (SPD-6Av). The mobile phase consisted of 80% methanol and 0.02 M sodium acetate buffer (60 : 40 v/v). The pH of the acetate buffer was 3.6. The column was heated to 40°C and wavelength of 320 nm was used. Calibration standards, controls, and samples were processed in batches. The stability of the samples under frozen conditions, at room temperature, and during freeze-thaw cycle was also determined. 

#### 2.2.12. Pharmacokinetic and Statistical Data Evaluation

The pharmacokinetic parameters were calculated using the Quick calk, computer PK calculation programme. The peak plasma concentration (*C*_max_) and time needed to reach peak plasma concentration (*T*_max_) were computed directly from plasma level profiles as a measure of the rate of absorption of the drug from each product. The elimination rate constant (*K*_el_) was calculated from the terminal elimination phase of logarithm of drug concentrations against time curve by the method of least square regression analysis. The biological halflife (*T*_1/2_) was determined by the relation:

(4) T1/2  =0.693K.

The extent of absorption for the drug (Microcid SR 75 mg capsule and formulation F_3_) in different subjects from the area under the plasma concentration time curve from zero to 24 h (AUC_0–24_) were calculated by the trapezoidal rule method. Area under the plasma concentration time curve from zero to infinity (AUC_0–*∞*_) was calculated using the formula;

(5) AUC0–∞=AUC0–T+C24K,

where *C*_24_ is drug concentrations in plasma at 24 h. The drug plasma concentration and pharmacokinetic parameters were analyzed by paired *t*-test and analysis of variance (ANOVA) at 95% confidence limit. Difference between two related means was considered statistically significant when their *P* values were equal to or less than 0.05.

## 3. Results and Discussion

 Evidence has [10–12] shown in the recent years that wax/fatty materials have the physical properties and behavior suitable to prepare gastroresistant, biocompatible, biodegradable microspheres to release the entrapped drug in the intestinal lumen [14, 15, 18]. In the present study, a modified novel meltable dispersion emulsified cooling-induced solidification method was employed using inert fat (CA) and nontoxic solvents to entrap the drug. In the present study, various parameters were studied such as drug and CA ratio, stirring speed and time, amount of surfactant added, volume of the aqueous phase used, effect of pH on drug entrapment, temperature of the aqueous phase, and rapid cooling studies. Therefore the influence of the above parameters was highlighted. When the pH value of the external aqueous phase was acidic, the solubility of the drug was reduced and the encapsulated amount of the drug increased. The maximum drug load was obtained at pH 4.2 (phthalate buffer). As the pH increased from 4.2 to 7.0, the percent of IM loading was reduced from 21.52 to 3.92%.

 In the present study, it was found that 150 mL of aqueous phase is suitable for producing the spherical microspheres. Resultant microspheres did not have any surface irregularities and are nonaggregated. As the volume of external phase increased, the yield was reduced and the resultant microspheres were irregularly shaped. When the volume of the aqueous phase was less than 150 mL, the resultant microspheres were highly aggregated in nature and highly impossible to distinguish as an individual microsphere. In order to avoid the formation of irregularly shaped larger particles, in the present method, 150 mL of aqueous phase was used. 

Incorporation of drug into CA microspheres required the addition of tween 80 as a surfactant, at an optimum concentration to reduce the interfacial tension between the hydrophobic material and external aqueous phase. An attempt was made to incorporate drug in the CA microspheres without the addition of a surfactant. But the process has a failed, as it resulted in an aggregate cake-like mass during the solidification of CA. This may be due to repulsion resulting from high interfacial tension between the hydrophobic CA material and external aqueous phase. It was found that tween 80 having an HLB value of 15 was suitable to increase substantially dispersion of CA in external aqueous phase and promote drug incorporation in the CA microspheres. To obtain an optimal surfactant concentration, various concentrations ranging from 0.1 to 0.6% (w/w) of the total formulation were tested. Discrete microspheres with good flow properties using an optimum concentration of surfactant 0.3% w/w (tween 80) were used. Concentrations of tween 80 ranging from 0.1 to 0.2% w/w failed to produce reproducible microspheres. The resultant CA microspheres were composed of irregular masses, which were not possible to distinguish as individual microspheres [10–12].

 Temperature of the aqueous phase was maintained at 5°C higher than the melting point of the CA in the corresponding formulations. From SEM studies it was observed that the resultant microspheres were free from surface irregularities, except some wrinkles. It was also observed that when the temperature of the aqueous phase was less than 5°C than the melting point of the CA, big flakes were produced.

In the present study, to produce the spherical discrete microspheres, an optimum drug to CA ratio of 1 : 3 w/w was used (Table 1). It was found that higher amount of drug to CA ratio (2 : 3) produces aggregate masses during the cooling process. It may be due to reduced melting point of the CA. SEM photographs also indicated the presence of the crystals on the surface of the microspheres. The resultant microspheres were unsuitable for pharmaceutical uses. Hence an optimum 1 : 3 ratio was used to prepare microspheres [10, 12, 14]. 

 It was observed that the average size of the microspheres ranged between 12 to 32 *μ*m as presented in Table 2. The important factor that influences the size distribution of microspheres is the optimum stirring speed and stirring time. A stirring speed of 900 rpm and stirring time of 3 min were used to obtain reproducible microspheres. It was observed that with the increase in the stirring speed from 900 to 1100 rpm there was a decrease in the average size of the spheres and recovery yield of the microspheres. It is due to small-sized wax microspheres, which were lost during successive washings. When the stirring speed was lower than 900 rpm, larger pellets were formed. It was also found that an increase in stirring time, from 2 to 4 min (at a stirring speed of 900 rpm), there was a decrease in the recovery yield of microspheres. When the stirring time was lower than 2 min, it was observed that some amount of melted material adhered to the sides of the beaker during the cooling process resulting in lower recovery of yield.

 Microparticulate drug delivery systems are formulated as single unit dosage forms in the form of capsule or tablet. Such microparticulate systems should possess the better and adequate micromeritic properties. The obtained micromeritic properties are given in Table 2. The values of angle of repose were well within the range, indicating reasonable good flow potential for the microspheres. The tapped density values ranged between 0.42 g/cm^3^ and 0.51 g/cm^3^. The results of % compressibility index ranged from 10.19% to 13.98%, suggests good flow characteristics of the microspheres (Table 2). The better flow property indicates reasonable and good flow potential of prepared microspheres. 

**Figure 1:**
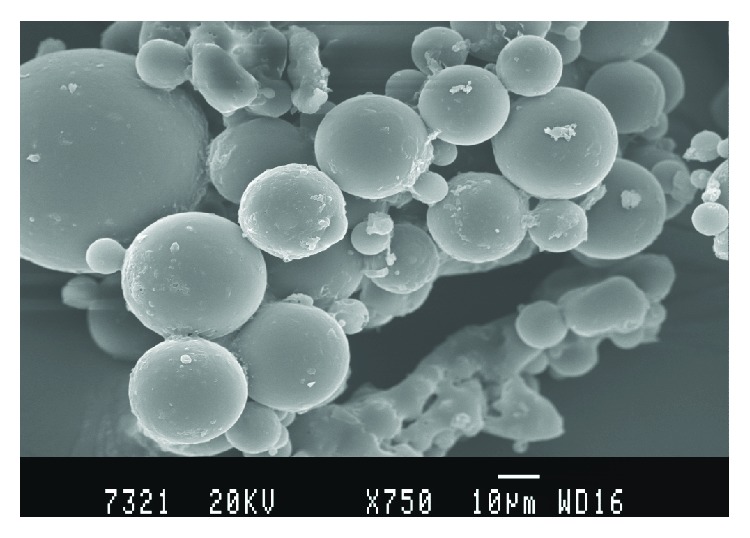
SEM photograph of cetyl alcohol microspheres loaded with IM-formulation F_3_.

 SEM photographs showed that the CA microspheres were spherical in nature and had a smooth surface with inward dents and shrinkage, which is due to the collapse of the wall of the microspheres (Figure 1). Microphotography reveals the absence of crystals of the drug on the surface of microsphere, indicating uniform distribution of the drug within the microspheres. The rate of solvent removal from the microspheres exerts an influence on the morphology of the final product. Interestingly, for microspheres dried at room temperature for 24 h, the sphericity values were nearer to the value 1, whereas for microspheres cured for 24 h at 4°C, the obtained sphericity values ranged between 1.13–1.19. The removal of residual moisture content from microspheres during curing exerts an influence on the morphology of the final product [15].

**Figure 2:**
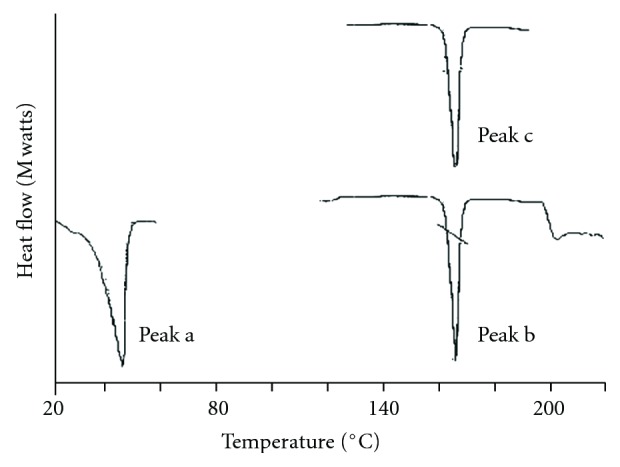
DSC thermograms of cetyl alcohol, pure indomethacin and indomethacin-loaded cetyl alcohol microspheres, peak a: cetyl alcohol, peak b: indomethacin, peak c: indomethacin-loaded cetyl alcohol microspheres (F_3_).

 DSC studies were performed on pure drug, empty, and drug-loaded microspheres, have shown sharp endothermic peaks. IM exhibits a sharp endothermic peak at 161.3°C presented in Figure 2. It was observed that presence of endothermic peak of the drug at 161.4°C in the drug-loaded CA microspheres indicates that the drug is uniformly distributed in the wall of the microspheres [14]. 

**Figure 3:**
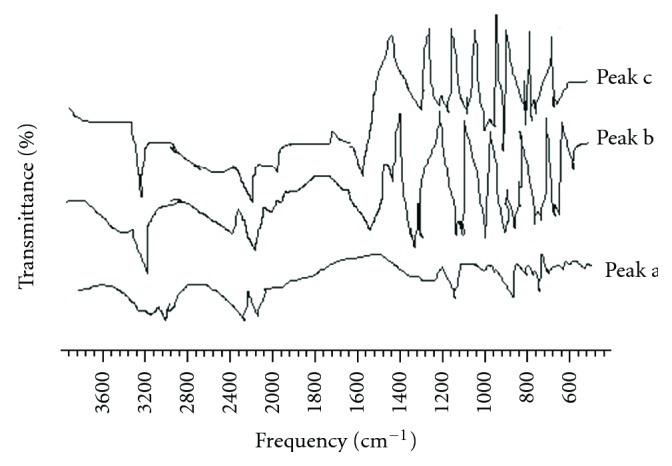
FTIR spectra of cetyl alcohol (peak a), indomethacin (peak b), and indomethacin-loaded cetyl alcohol microspheres (peak c:F_3_).

 FTIR spectra for IM and formulation F_3_ are shown in Figure 3. The characteristic IR absorption peaks of IM comparing the IR spectra at 3413 (aromatic C–H stretching), 2618 (carboxylic acid stretching), 1695 (C=O stretching), 1600 (C=C stretching), 1452 (O–CH_3 _deformation), and 1236 cm^−1^ (O–H deformation) were not altered after successful encapsulation of drug, indicating no chemical interactions between the drug and CA used. A comparison and an interpretation of this region in our spectra agree with their conclusions [14, 18]. 

 The percent of drug loading in the formulations was found to be in the range of 18.67–21.52%. It was low in the formulation F_4_ (19.46) and more in F_3_ (23.52). 

 The encapsulation efficiency (%) was found to be more in formulation F_3_ (94.34%) as compared to F_1_ (89.54%), F_2_ (89.63%), F_5_ (88.98%), and F_4_ (87.67%). From this result, it can be concluded that the formulation F_3_ had more encapsulation efficiency. 

**Figure 4:**
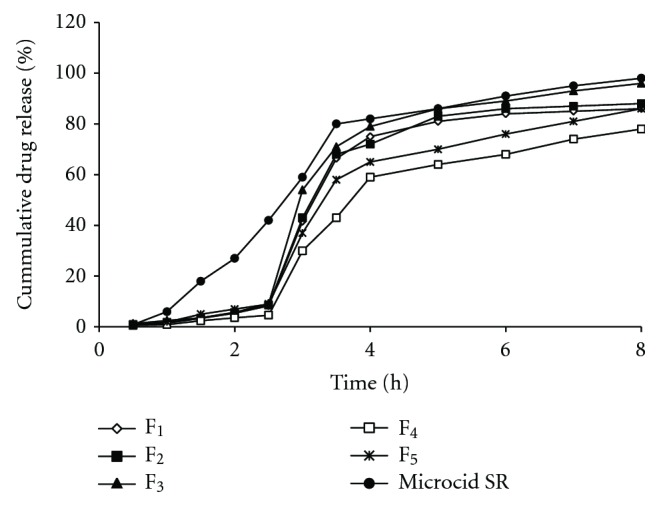
Drug release profile of indomethacin from microspheres and Microcid SR.

From the release studies depicted in Figure 4, it was observed that there is no significant release of drug at gastric pH from fat microspheres. At the end of 8th h, *in vitro* drug release from F_3_ (96.32%) was slower than Microcid SR (98.98%) in the intestinal environment. Drug was released in a biphasic manner consisting of initial fast release followed by a slow release in intestinal pH from the CA microspheres [2, 10, 12, 18]. The decreased *in vitro* drug release from CA microspheres might be due to more hydrophobicity and influence of molecular weight of CA. *In vitro* drug release was considerably retarded from the CA microspheres when compared to Microcid SR. The rate of drug release followed first order release kinetics and numerical data fitted into Peppas model showed that the mechanism of drug release from CA microspheres was fickian diffusion (F_1_-0,412_, _F_2_-0.415_,_ F_3_-0.398_,_ F_4_-0.421, F_5_-0.431, and Microcid SR-0.476). After an initial burst effect, the subsequent release of drug from microspheres was slow.

 Microcid SR 75 mg capsule and formulation F_3_ were subjected for stability studies for 90 days. It was observed that *in vitro* drug release from Microcid SR 75 mg capsule and formulation F_3_ at the end of 90 days (8th h) were 99.43 and 95.09%, respectively. However, no significant change in *in vitro* drug release from both the products was noticed after the study period, indicating good stability for the prepared formulation. 

Drug content uniformity for Microcid SR 75 mg capsule and formulation F_3_ was found to be 74.83 mg and 74.80 mg, respectively. The percents of drug content uniformity of Microcid SR 75 mg capsule and formulation F_3_ are 99.76 and 99.49%, respectively. Hence, the percent of drug content uniformity in both products were well within the limits as per United State Pharmacopoeia and National formulary specification [16].

 Recovery of the IM from the plasma was calculated by comparison of peak height ratio after direct injection of IM or MA to the peak height of the same concentrations of the analytes extracted from plasma. In both cases the absolute IM recovery from plasma was over 91%. The extraction solvent selected in this investigation gave higher recoveries and clean extracts than other solvents tested. Plasma spiked with 500 ng/mL of IM and 1000 ng/mL of MA the retention times for IM and MA were 5.52 and 8.23 min, respectively. Sensitivity of HPLC assay qualitative confirmation of the purity of IM and MA peaks were obtained (Table 3). The limit of quantification was 50 ng/mL of IM in plasma when 0.5 mL plasma was placed. The obtained mean correlation coefficients for the standard curves (*n* = 6) was 0.998. Assay was shown to be sensitive, capable of reliably detecting IM concentrations in plasma as low as 50 ng/mL. Interferences from endogenous compounds were overcome by using an acidic buffer (citrate buffer pH 3.0) to alter the pH of the aqueous phase before extraction. To prevent the substantial interferences from endogenous compounds, strong acid-like HCl was employed.

**Figure 5:**
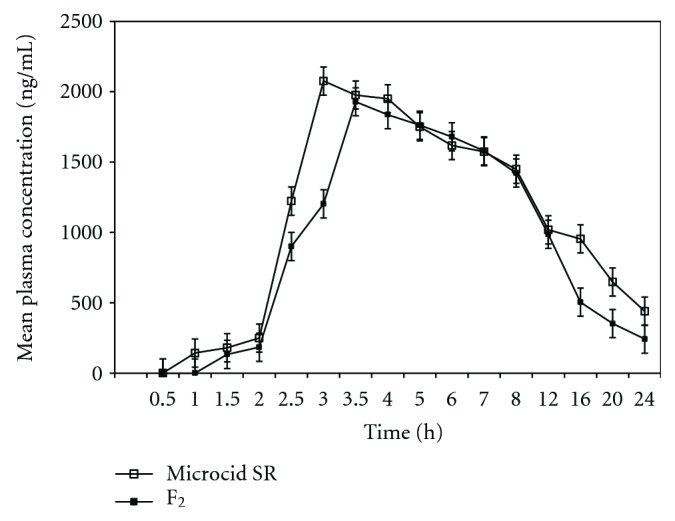
Mean plasma concentrations:time profiles of indomethacin from Microcid SR and formulation F_3_.

The mean plasma concentration as a function of time is shown in Figure 5 and the calculated pharmacokinetic parameters of Microcid SR and F_3_ formulations are given in Table 4. After oral administration of both products, more mean *C*_max_ value was observed for Microcid SR 75 mg capsule (2134  ±  29.6 ng/mL) than formulation F_3_ (1989  ±  20.30 ng/mL). However, the difference in the *C*_max_ values obtained for Microcid SR 75 mg capsule and formulation F_3_ was statistically insignificant. Mean plasma concentrations of IM for both products in all experimental conditions were within the therapeutic concentration range (300–3000 ng/mL) [9]. The *C*_max_ values for both products do not exceed the above limit in all animals. It was observed the plasma concentration of IM falls below detection limit (50 ng/mL) after 24 h in all animals following administration of either product. On the basis of the therapeutic concentration range of IM, it could be concluded that the therapeutic effects of both formulations would be probably be maintained for about 12 h following a single dose administration. Thus it could be predicted that the two controlled release formulations included in this study are associated with a similar onset of therapeutic response following a single dose administration under fasting conditions. Furthermore, it could be predicted that both controlled release formulations in this study are associated with a similar onset of therapeutic response, following a single dose administration under fasting conditions.

The time taken to reach peak plasma concentration *T*_max_ of IM was little higher in case of Microcid SR compared to formulation F_3_, but no statistical significance differences between two products are observed (Table 4). The calculated mean *T*_1/2_ values for Microcid SR and formulation F_3_ were 2.62  ±  0.02 h^−1^ and 2.67 ± 0.02 h^−1^, respectively. There was not much difference in the *T*_1/2_ for IM between both formulations, and no statistical significance differences were observed between both products. Mean rate of absorption *K*_*a*_ for Microcid SR was 0.3934  ±  0.002 h^−1^ and for formulation F_3_0.3856  ±  0.002 h^−1^ and mean elimination rate constants *K*_el_ for Microcid SR and for formulation F_3_ were 0.2843  ±  0.004 h^−1^ and 0.2678  ±  0.004 h^−1^, respectively.

However a small difference between both products related to *C*_max_, *T*_max_, *T*_1/2_ and reduced fluctuations (peak to trough ratios) of the plasma concentrations. All these effects probably may be due to the dissolution rate limited drug release and hence absorption. From the study it was observed that reduced fluctuations combined with the elevated mean plasma concentration from both products offer advantage in protecting patients against morning stiffness [18]. 

 The systematic availability of IM can be determined by comparison of the area under the plasma concentration (AUC) versus time curves. The mean AUC_0–24_ values for Microcid SR and formulation F_3_ were 12478  ±  104.21 ng/mL h^−1^ and 12145  ±  87.32 ng/mL h^−1^, respectively. Slow *in vitro* release of IM from the products Microcid SR and F_3_ formulations may be responsible for the decreased AUC values when compared to the reported conventional dosage forms [19]. The average value of the individual and mean AUC_0–24_ ratio at 95% confidence limit (0.8–1.25) was within acceptable limits for bioequivalent products [19]. In order to obtain *in vitro-in vivo *correlation, drug absorption profiles were compared for Microcid SR and formulation F_3_ using the cumulative fraction of the drug absorbed *in vivo* against cumulative fraction of the drug dissolved *in vitro* up to 8 h. From the study it was noticed that both products showed an adequate correlation. Currently accepted criteria in the US for bioequivalence for most dosage forms require that the mean pharmacokinetic parameters of the test dosage forms should be within 80–120% of the reference dosage form using 90% confidence interval. Pharmacokinetic parameters clearly indicate that the parameters of F_3_ are in good agreement with Microcid SR. The observed mean AUC_0–*∞*_values for Microcid SR and formulation F_3_ were 12632  ±  132.12 ng/mL.h^−1^ and 12452  ±  96.32 ng/mL.h^−1^ which does not show any significant statistical difference between the products.

On the basis of FDA recommendation [20], the two products, Microcid SR and formulation F_3_, can be considered bioequivalent. No untoward effects were observed by any of the subjects after the administration of either product. Thus, the two formulations can be considered similar, because all the subjects are very well tolerated. These observations clearly indicate the absence of high peak plasma concentrations (>5000 ng/mL), which are very often associated with adverse effects due to drug accumulation [8], because of the accumulation effect. The products Microcid SR and formulation F_3_ investigated in the present study were found to be bioequivalent. 

## 4. Conclusion

The objective of the study was to prepare and evaluate wax microspheres loaded with IM by optimized meltable dispersion emulsified cooling-induced solidification method for controlled release. The method employed was simple, rapid, and economical and does not imply the use of toxic organic solvents. The results of the drug entrapment and micromeritic properties exhibited fairly good spherical nature as evidenced by SEM photograph. The compatible state of the drug-loaded wax microspheres was evaluated by FTIR and DSC. Both formulations were found to be bioequivalent and both formulations showed an adequate correlation between cumulative fractions dissolved *in vitro* and cumulative fractions absorbed *in vivo*. Optimized formulation F_3_ and marketed product Microcid SR showed similarity in drug release profiles and *in vivo* bioequivalent behavior. From the present work, it can be concluded that the prepared wax microspheres demonstrate the potential use of cetyl alcohol for the development of controlled drug delivery systems for water insoluble or lipophilic drug.

## Figures and Tables

**Table 1:** Drug and cetyl alcohol ratio for the prepared microspheres formulations.

Formulation	Drug (gm)	Cetyl alcohol (gm)
F_1_	2.8	8.8
F_2_	2.9	8.9
F_3_	3.0	9.0
F_4_	3.1	9.1
F_5_	3.2	9.2

**Table 2:** Micromeritic properties of the drug-loaded CA microspheres.

Formulation	Average size (*μ*m)∗	Yield (%)∗	Angle of repose (*θ*°)∗	% compressibility index∗	Tapped density (g/cm^3^)∗
F_1_	12 ± 02	91.43 ± 1.2	25.32 ± 1.6	10.19 ± 0.8	0.42 ± 0.3
F_2_	14 ± 01	93.11 ± 1.3	26.98 ± 1.3	10.89 ± 0.5	0.44 ± 0.4
F_3_	25 ± 03	95.88 ± 0.9	27.43 ± 1.0	12.12 ± 0.9	0.51 ± 0.5
F_4_	28 ± 03	90.21 ± 1.1	24.98 ± 1.3	13.67 ± 0.7	0.48 ± 0.4
F_5_	32 ± 04	90.10 ± 1.0	25.12 ± 1.1	13.98 ± 0.6	0.49 ± 0.5

∗Mean ± standard deviation, *n* = 3.

**Table 3:** Absolute recovery results obtained for indomethacin from plasma.

Sampling Time (h)	Drug present in ng/mL A	Drug added in ng/mL B	Drug recovered in ng/mL C	Drug Conc. recorded in ng/mL C–A	Percent of drug recovered C–A × 100/B Mean ± SD∗
0.5	10	50	58	48	96.0 ± 1.31
2.0	10	100	106	96	96.0 ± 1.24
4.0	10	200	207	197	98.5 ± 1.42
6.0	10	300	308	298	99.33 ± 1.12
8.0	10	400	406	396	99.00 ± 1.54

∗Standard deviation, *n* = 5.

**Table 4:** Comparison of mean values of pharmacokinetics obtained for products Microcid SR and formulation F_3_ after oral administration.

Parameters∗	Microcid SR	Formulation F_3_	*P* value
*T*_max_ (h)	3.0	2.9	>0.05
*C*_max_ (ng/mL)	2134 ± 29.60	1989 ± 20.30	>0.05
*T*_1/2_ (h^−1^)	2.62 ± 0.02	2.67 ± 0.20	>0.05
AUC_0–24_ (ng/mL h^−1^)	12478 ± 104.21	12145 ± 87.32	>0.05
AUC_0–*∞*_ (ng/ML h^−1^)	12632 ± 132.12	12452 ± 96.32	>0.05
*K*_a_ (h^−1^)	0.3934 ± 0.002	0.3856 ± 0.002	>0.05
*K*_el_ (h^−1^)	0.2843 ± 0.004	0.2678 ± 0.004	>0.05
Mean residence Time (MRT)	4.89 ± 0.03	4.75 ± 0.03	>0.05

∗Mean ± standard deviation, *n* = 3.
